# Influential Pathways of Employees’ Career Growth: Linkage of Psychological and Organizational Factors Based on Qualitative Comparative Analysis

**DOI:** 10.3389/fpsyg.2021.796454

**Published:** 2022-01-04

**Authors:** Bailin Ge, Zhiqiang Ma, Mingxing Li, Zeyu Li, Ling Yang, Tong Liu

**Affiliations:** ^1^School of Management, Jiangsu University, Zhenjiang, China; ^2^Xinjiang Enterprise Development Research Center, School of Business Administration, Xinjiang University of Finance and Economics, Ürümqi, China; ^3^Jingjiang College of Jiangsu University, Zhenjiang, China; ^4^Huashanwan Community Health Service Station, Zhenjiang, China; ^5^Office of Academic Affairs, Zhenjiang College, Zhenjiang, China

**Keywords:** employees career growth, entrepreneurial psychological factors, organizational factors, configurational theory, influential pathways

## Abstract

Implementing the “hierarchical diagnosis and treatment” system highlights the important role of general practitioners as “residents’ health gatekeepers.” Still, the low level of career growth always limits the realization of their service value. Inertial thinking uses a single factor to explain the complexity of career growth in previous studies; in fact, it isn’t easy to assess whether the factor is a sufficient and necessary condition for a high level of career growth. Herein, we have used a set theory perspective to analyze the mechanism of influencing high-level career growth by combining psychological and organizational factors. This research aims to analyze causal complexity relationship between these conditions and results is analyzed in detail. We choose fuzzy-set qualitative comparative analysis (fsQCA) with a sample of 407 GPs to test 5 antecedent conditional variables that can affect their career growth. The variables include professional identity, self-efficacy, achievement motivation, training mechanism, and incentive mechanism. To ensure the universality and diversity of data, the samples were selected from community medical institutions in different regions of China. The results show that three pathways can affect the high career growth of GPs, and the optimal pathway A2 is the linkage matching of high incentive mechanism, high professional identity, high achievement motivation, and high self-efficacy. At the same time, we find that professional identity plays an alternative role in the three pathways. When professional identity is at a high level, as long as achievement motivation and self-efficacy are superior, or achievement motivation, self-efficacy, and achievement motivation are superior, a high level of career growth can be achieved. We broke the shackles of previous studies that only focused on the impact of single factors on the career growth of GPs. From the perspective of set theory, we use configurational thinking to construct Influential pathways of high career growth of GPs by integrating antecedents. The results can provide effective support for improving GPs’ service ability and realizing their service value to protect residents’ health.

## Introduction

With the rapid development of the economy and the continuous improvement of living standards, the residents’ demands for health are getting higher and higher. However, the huge population is gradually showing the trend of an aging population and the prevalence of diseases in China (Yang et al., 2019; Zhang et al., 2021), which puts forward higher medical and health services requirements (Liu et al., 2019). How to protect and improve the health of residents is becoming an urgent issue. According to the actual situation, the government proposed the policy of “serious diseases into hospitals and minor diseases into communities” to realize the medical service system of community first diagnosis. On the one hand, it can alleviate the pressure of medical treatment in large hospitals (Meng and Pan, 2013). On the other hand, it can promote the development of community health service medical system (Liu et al., 2019; Zhou et al., 2020). As the core force of community development, general practitioners (GPs) play an important role as “residents’ health gatekeepers,” however, the low professional ability, the vague career goal development planning, and the lack of attention to humanistic care greatly hinder the process of their career growth (Wang et al., 2019), resulting in a serious mismatch between their lower service ability and their responsibilities (Zhang et al., 2020). It has formed such a situation that the GPs have not trusted the residents, and the community’s first diagnosis policy is difficult to promote.

Career growth is a dynamic process, reflecting the direction and speed of the flow of individual professional values that influence practices (Hui and Graen, 1997). According to the guidance of the State Council of China, general practitioners are defined as comprehensive medical talents who can integrate prevention, health care, diagnosis, treatment of common/frequently occurring diseases and referral, patient rehabilitation, and chronic disease management (Liu et al., 2018). So, they are called the “residents’ health gatekeepers” and show the characteristics of continuous and comprehensive medical services. Combined with the above definition, the career growth of GPs can be described as the process in which individuals develop along with more valuable career goals, continuously improve their professional ability, apply more emotions to their work, and bear more responsibilities and accept more challenges with the accumulation of experience.

The key to promoting the development of GPs’ career growth is to explore its influencing factors deeply. The government has developed a training mechanism and incentive mechanism to enhance GPs’ professional ability and mobilize their working enthusiasm to accelerate GPs’ career growth. To improve the training quality of GPs, strengthen the construction of training bases and teaching staff, the National Health Commission and the education department have improved the training system and applied various training methods for GPs, such as standardized training, job-transfer training, directional free training and so on. In the document named “Opinions on reforming and improving the incentive mechanism of training and using general practitioners” issued by the General Office of the State Council, PRC on January 24, 2018, many preferential measures have been put forward for the career development of GPs based on salary and career planning, which has enhanced the sense of professional honor and adhered to the combination of spiritual rewards and material rewards. At present, most researchers are focusing on improving career growth and have summarized the correlated influencing factors from multiple perspectives (Ellis et al., 2020; Harris et al., 2020; Zhao et al., 2020). In general, career growth is a dynamic development process that is influenced by values and systems, divided into psychological and organizational levels.

At the psychological level, scholars mainly emphasize the important influence of professional identity (PI), achievement motivation (AM), and self-efficacy (SE) on career growth and have conducted in-depth studies. PI is the degree of consistency between the individual and his occupation or the perception of belonging to a certain occupation (Johnson et al., 2006), which is the basic element for realizing the value of life. The fundamental driving force for promoting GPs’ career growth lies in the recognition and devotion of their role as “residents’ health gatekeepers” (Johnson et al., 2006; Gu et al., 2019). The higher the PI, the greater the degree of responsibility and efforts of GPs for this profession, while the low PI will lose professional confidence and easily lead to job burnout (Li L. et al., 2020). AM is the desire to achieve a certain goal through efforts, which reflects the individual’s preference for success, and it is the internal driving force to pursue success. The results of career growth depend on self-motivation and preference for success, and high AM easily enhances the desire to develop a high level of professional ability and accelerates the achievement of career goals (McClelland, 1976; Liu et al., 1991; Consoli et al., 2010). Generally, GPs with high AM have a strong professional ability, so they are more likely to be recognized and trusted in helping patients relieve pain and health guidance. Significantly, this sense of honor will stimulate their motivation to succeed, so as to achieve high career growth. SE is the judgment and self-confidence in one’s ability to achieve a specific goal when facing difficulties (Bandura and Locke, 2003). SE is an important internal driving factor that can influence human behaviors and act as a lasting driving force for promoting career growth (Yao et al., 2013; Yoon and Christopher, 2016). GPs with high SE have a serious work attitude, stable mood, clear logic, and strong communication skills. They improve their skills and purify professional ethics to achieve high professional goals and professional ability.

The training mechanism (TM) and incentive mechanism (IM) have profound influences at the organizational level and have been subject to extensive research on career growth. For GPs, training can improve professional skills and humanistic qualities (Berkhof et al., 2011; Clayton et al., 2013; Stephen and Robert, 2016; Wang et al., 2017). The process of personal career growth needs to be recognized by organizations (Stephen et al., 2015; Laurence et al., 2016). Hence, the IM can guide and control individual behavior to mobilize enthusiasm (Campion et al., 2011; Peterson et al., 2019; Li X. et al., 2020), an effective driving force for GPs to achieve their career goals. Specifically, material incentives should ensure that the basic living needs of GPs can be satisfied (He and Long, 2011). Mental stimulation can produce strong emotional dependence, allowing GPs to obtain additional happiness and identity from work (Zhang et al., 2014). In summary, high TM and IM can promote and stimulate the realization of GPs’ professional goals, the improvement of professional ability and emotional investment.

According to the summary of the existing articles, we find a certain correlation between psychological and organizational factors. The scholar believes that the SE of enterprise managers is positively correlated with AM (*r* = 0.55, *P* < 0.01) (Yang and Gu, 2007). The PI of undergraduate nursing students is positively correlated with AM (*r* = 0.42, *P* < 0.01), and negatively correlated with the motivation of avoiding failure (*r* = 0.38, *P* < 0.01) (Jiang et al., 2014). The PI of male nurses has a positive impact on learning SE (*r* = 0.28, ∼ 0.35, *P* < 0.01) (Zuo et al., 2011). It is found that there is a positive correlation1 between PI, AM, and SE, and the three factors can predict each other. It has been reported that improving the salary and welfare level of GPs can enhance their professional self-confidence and PI (Zhang et al., 2014). Regular training can effectively improve the professional identity, self-efficacy and achievement motivation of GPs (Jiang and Zhou, 2019). Therefore, psychological and organizational factors have the basic conditions for linkage.

**FIGURE 1:**
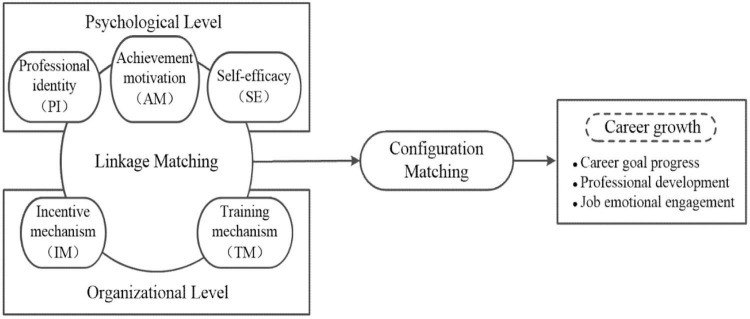
Career growth driving mechanism model.

However, the current research mainly focuses on a single level and does not comprehensively evaluate the multiple driving factors affecting the high career growth of GPs. The internal mechanism of collaborative interaction between the psychological and the organizational level affecting the career growth of GPs is relatively vague, and the influence pathways of multiple concurrent factors are ignored. The career growth of GPs is a complex process affected by the synergy of psychology and organization. Qualitative comparative analysis (QCA) uses the perspective of configuration analysis to explore and integrate the complexity of antecedent conditions, which can fully combine the advantages of quantitative and qualitative research (Yan et al., 2018; Du et al., 2021). As shown in Figure 1, five antecedent conditions, including PI, AM, and SE at the psychological level and IM and TM at the organizational level, are integrated to form a variety of configurations *via* QCA. Finally, based on the analysis of the reliability and validity of the research data, we conducted fuzzy set processing (calibration) and necessary condition analysis on the data according to the steps of FsQCA, so as to further analyze the configurations of high and non-high career growth of GPs.

## Materials and Methods

### Sample

The data were collected in the form of online questionnaires between May and June 2020 on the Questionnaire Star platform. The questionnaires covered 20 provinces, four municipalities, and five autonomous regions by simple random sampling in China. The types of medical institutions of GPs included hospitals, community health service centers (stations), central health hospitals, township (town) health hospitals, village health clinics (stations), and clinics. Finally, 407 valid responses were obtained from 465 distributed questionnaires, yielding a validity rate of 87.53%. The sample covers hundreds of medical institutions in most provinces and cities of China (almost all types of medical institutions where GPs work). From the perspective of gender characteristics of the sample, the proportion of men and women is not much different, and the proportion of women is slightly higher, accounting for 52.09%. From the working years, age structure, education level, and position of the sample, 60.93% of the GPs have more than 10 years of working experience, 62.41% are over 40 years old, 69.29% have bachelor’s degree, 10.32% have a graduate degree, and 93.36% have a senior professional title, which indicates that most of the GPs in the sample have senior working experience and high cultural quality. From the perspective of team size, 82.31% of the teams have more than 15 people, which indicates that most of the GPs in the sample has a large team size. Generally, the development of medical teams of this size is relatively mature and representative. The basic characteristics of the research sample are shown in Table 1.

**TABLE 1:** Basic characteristics of the research sample.

GP characteristics	Measurement items	Sample size	Percentage (%)
Gender	Male	195	47.91
	Female	212	52.09
Age	21–25 years old	14	3.44
	26–30 years old	44	10.81
	31–35 years old	40	9.83
	36–40 years old	55	13.51
	Over 40 years old	254	62.41
Working years	Less than 1 year	29	7.13
	1–3 years	39	9.58
	3–5 years	36	8.85
	5–10 years	55	13.51
	More than 10 years	248	60.93
Education level	High school and below	9	2.21
	Junior college	74	18.18
	Undergraduate	282	69.29
	Postgraduate	42	10.32
Team size	Fewer than 5 people	35	8.6
	6–10 people	17	4.18
	11–15 people	20	4.91
	15 people or more	335	82.31
Position	Medical assistant	27	6.63
	Resident	65	15.97
	Attending physician	139	34.15
	Others (chief physician, deputy chief physician, practicing assistant physician, etc.)	176	43.24

### Fuzzy-Set Qualitative Comparative Analysis Procedures, Methods, and Data Preparation

The American sociologist Ragin first proposed qualitative comparative analysis (QCA) in the 1980s (Ragin, 1987). The method aims to solve causal complexity and emphasizes that it is not just a single factor that affects the results. Significantly, from a holistic perspective, different pathways affecting the results are often obtained by exploring a combination of antecedent conditions. A new causal relationship was developed based on the QCA method—“Multiple concurrent causalities.” The QCA method denies any form of constant causality and holds that the causality depends on the specific situation and configuration (Du and Phillip, 2021). Therefore, it is different from the mainstream statistical method of developing a single causal model that best fits the data. Instead, it focuses on the diversity and complexity of causality and determines the number and characteristics of different causal models among multiple comparable cases (Medina et al., 2017). QCA makes up for the deficiency of qualitative analysis and quantitative analysis. It not only conducts statistical analysis on the research object but also analyzes the whole research object. It can deal with a few case studies and solve the problems of causal complexity in a large number sample by configurational analysis.

In our study, 407 GPs included in a previous large-sample study were investigated (Du and Jia, 2017). However, to avoid the complication of the research results caused by too many conditions (Greckhamer et al., 2013), we selected the standard of 4–7 condition variables concerning the medium sample (10–50). We set five condition variables (professional identity, achievement motivation, self-efficacy, incentive mechanism, and training mechanism), they were selected to analyze the outcome variable (career growth). QCA mainly includes crisp-set QCA (csQCA) and fuzzy-set QCA (fsQCA). Herein, fsQCA was used for data analysis because of its universality and practicability. Specifically, fsQCA is based on “set theory” to analyze further the necessity and sufficiency of antecedent conditions and conduct condition configuration analysis.

### Measures and Calibrations for Set Membership

All variables were measured with a 5-point Likert scale. The three dimensions of career growth of GPs included progress toward vocational goals, development of professional ability, and emotional engagement in their positions, which was obtained *via* the form of semi-structured interviews (face to face) with 38 GPs (from 11 community hospitals in Zhenjiang City, Jiangsu Province) based on the grounded theory. Then, based on the career growth research scale developed by Weng and Hu (2009), a 12-item questionnaire was designed (an example item is “Basic medical work such as disease management has brought me closer to my career goals”). The results showed that Cronbach’s alpha coefficient was 0.740, the AVE was higher than 0.5, and the CR was higher than 0.7. The minimum square root of 0.722 was greater than the maximum correlation coefficient of 0.114. Thus, the questionnaire showed good internal consistency reliability, content validity, and discrimination validity.

The single-dimension professional identity (PI) scale developed by Wei (2008), combined with the college teacher PI scale developed by Zhu (2012), was used in this study. There were five items in total (e.g., “I am proud of being a general practitioner”).

The single-dimension achievement motivation (AM) scale developed by Zhou (2008), combined with the professional AM scale on science and technology talents developed by Wang et al. (2020), was applied in this study. There were five items in total (e.g., “I am willing to be challenged by more difficult tasks”).

The single-dimension self-efficacy (SE) scale that was developed by Schwarzer et al. (1999) and translated and revised by Wang et al. (2001) was used in this study. There were six items in total (e.g., “I believe I can deal with unexpected things”).

The four dimensions of the incentive mechanism (IM) scale summarized *via* the scale developed by Mao (2012) and the working characteristics of GPs were used in this study. There were four items total (e.g., “My unit will give us some kind of medal or honorary title”).

The three dimensions of the training mechanism (TM) scale summarized *via* the training mechanism model developed by Liu (2013) and the Kirkpatrick training evaluation model were adopted in this study. There were six items in total (e.g., “The content and training methods are consistent with the job requirements”).

In Table 2, the reliability and validity of each variable are presented. The Cronbach’s alpha coefficients of all variables were greater than 0.7 (O’Leary-Kelly and Vokurka, 1998), indicating that the variables had excellent reliability. The factor loadings were all higher than 0.6, reflecting that the variables had outstanding convergent validity. Furthermore, the internal consistency reliability was greater than 0.75, and the AVE was greater than 0.5, indicating good discriminant validity among the variables.

**TABLE 2:** Reliability and validity analysis results.

Variable	Dimension	Measurement standard	Loading	Cronbach’s alpha coefficient	AVE	Internal consistency reliability
Career growth	Career goal progress	S1	0.775	0.841	0.640	0.842
		S2	0.849			
		S3	0.773			
	Professional development	S5	0.633	0.867	0.522	0.867
		S6	0.723			
		S7	0.736			
		S8	0.793			
		S9	0.727			
		S10	0.729			
	Job emotional engagement	S12	0.713	0.784	0.556	0.788
		S13	0.812			
		S14	0.700			
Professional identity	–	S15	0.797	0.899	0.689	0.913
		S16	0.908			
		S18	0.861			
		S19	0.602			
		S20	0.844			
Achievement motivation	–	S21	0.655	0.821	0.516	0.837
		S22	0.766			
		S23	0.753			
		S24	0.691			
		S25	0.725			
Self-efficacy	–	S26	0.655	0.901	0.627	0.907
		S27	0.775			
		S28	0.825			
		S29	0.887			
		S30	0.876			
		S31	0.651			
Incentive mechanism	–	S32	0.807	0.894	0.680	0.895
		S33	0.809			
		S34	0.846			
		S35	0.836			
Training mechanism	–	S36	0.830	0.946	0.750	0.947
		S37	0.832			
		S38	0.886			
		S39	0.886			
		S40	0.911			
		S41	0.853			

In our study, the “direct method” was used to calibrate the original data. According to the measurement values of the 5-point Likert scale, we assigned the scale anchors 95, 50, and 5% as the thresholds for fully in, crossover, and fully out. The assignment criteria are shown in Table 3, and the calibrated sets were subordinate to 0–1. We conducted a sufficiency analysis following established QCA procedures by using a frequency benchmark ≥ 2, raw consistency benchmark ≥ 0.8, and a proportional reduction in inconsistency (PRI) ≥ 0.70 (Greckhamer et al., 2018).

**TABLE 3:** Fuzzy set calibrations.

Research variables		Threshold value		
		Fully out	Crossover	Fully in
Conditional variables	Professional identity (PI)	2.98	3.9	4.7
	Achievement motivation (AM)	3.14	4	4.7
	Self-efficacy (SE)	3.2	4	4.7
	Incentive mechanism (IM)	3	3.9	4.7
	Training mechanism (TM)	2.94	3.9	4.7
Outcome variable	Career growth	3.3	4.1	4.7

## Results

### Necessary Conditions Analysis

The necessary conditions needed to be tested before constructing the truth table (Supplementary Table 1). We conducted a fuzzy-set analysis of necessary conditions using a consistency benchmark of 0.90, the antecedent conditions exceeding the benchmark were considered a superset of the result variables. As shown in Table 4, no single variable was necessary for high career growth. Therefore, the antecedent conditions were entered in fsQCA to explore the configurations of high career growth.

**TABLE 4:** Analysis of necessary conditions for GPs career growth in FsQCA.

Sets of condition	Outcome variable	
	High career growth	Not-high career growth
Professional identity	0.899	0.532
∼Professional identity	0.489	0.843
Achievement motivation	0.854	0.506
∼Achievement motivation	0.519	0.845
Self-efficacy	0.842	0.516
∼Self-efficacy	0.513	0.819
Incentive mechanism	0.839	0.550
∼Incentive mechanism	0.518	0.785
Training mechanism	0.797	0.524
∼Training mechanism	0.554	0.806

### Configuration Analysis

The fsQCA method can obtain three kinds of solutions: complex, intermediate, and parsimonious. The difference between these solutions lies in the configurational types included in the analysis scope when conducting Boolean minimization analysis. As the complex solutions are not simplified, more configurations are obtained, which is not conducive to analyze the result pathways. The parsimonious solution takes all the logical remainder into the simplification process, and it is easy to simplify the important necessary conditions. However, an important advantage of the intermediate solution is that it does not eliminate the necessary conditions. It retains the necessary conditions and can explain the configurational mechanism (Rihoux and Ragin, 2009). Therefore, the intermediate solution should be illustrated in QCA research (Zhang and Du, 2019).

The difference between the core and peripheral conditions lies in whether the antecedent conditions appear in parsimonious and intermediate solutions. If the antecedent condition exists, it is called the core condition and has an important influence on the result. If the antecedent condition only appears in the intermediate solutions, it is called a peripheral condition and plays an auxiliary role (Fiss, 2011).

According to the analysis of necessity, the single antecedent variable was weak in explaining the career growth of GPs. Therefore, five antecedent conditions were analyzed through fsQCA 3.0 to obtain the combination of antecedent conditions of career growth. According to the results, we analyzed psychological and organizational factors’ influence pathways on career growth.

After processing the calibration results of 407 cases *via* fsQCA 3.0, we obtained three solutions, including complex, parsimonious, and intermediate. In Table 5, the intermediate solution was obtained based on the counterfactual analysis. It was assumed that the appearance of each condition variable could promote the career growth of GPs. According to the fsQCA method, three configurations (A1, A2, and A3) could produce high career growth, and the consistency indexes of the three configurations were 0.882, 0.866, and 0.855, respectively. The three configurations are all-sufficient conditions for high career growth. The consistency of the model solution is 0.855, which further indicates that the three configurations covering the majority of cases are sufficient conditions. The coverage of the solution is 0.861, indicating that they explain about 86% of the reasons for high career growth. Simultaneously, assuming that the absence of each condition variable may lead to not-high career growth, the fsQCA method shows that there are two configurations (NA1, NA2), and they cover the vast majority of cases, not only constituting sufficient conditions but also explaining the reasons for about 83% of not-high career growth.

**TABLE 5:** Configurations for achieving GPs career growth (FsQCA).

Antecedent conditions	High career growth			Not-high career growth	
	A1	A2	A3	NA1	NA2
Professional identity (PI)	•	•	•		•
Achievement motivation (AM)	•	•	⊗	⊗	•
Self-efficacy (SE)	•	•	⊗	⊗	•
Incentive mechanism (IM)		•	⊗	⊗	
Training mechanism (TM)	⊗		⊗	⊗	⊗
Consistency	0.882	0.866	0.855	0.869	0.831
Raw coverage	0.436	0.808	0.416	0.756	0.387
Unique coverage	0.003	0.380	0.045	0.395	0.026
Overall solution coverage	0.861			0.782	
Overall solution consistency	0.855			0.833	

*“•” indicates the existence of core causal conditions, “⊗” indicates the absence of core causal conditions, “•” indicates the existence of peripheral conditions, and “⊗” indicates the absence of peripheral conditions.*

### High Career Growth Configurations of General Practitioners

(1) A1: PI*AM*SE*∼ TM

Regardless of whether the TM is perfect, as long as there is a high PI, clear AM, and strong SE, the career growth behavior of GPs will be triggered. PI, AM, and SE are interconnected internal driving forces. High PI is the premise of achieving high AM and high SE. In detail, for GPs, the higher recognition of their role as “residents’ health gatekeepers,” the more efforts they will make to reduce disease incidence rates among patients and provide primary care services in the community, which will further strengthen their belief during the process of improving the ability to face challenges. In contrast, the more self-confident they are in achieving their established goals (possessing higher SE), the more motivated they will be to succeed. Simultaneously, they are willing to make more efforts to improve their professional ability and enrich their professional emotions (Wang et al., 2014; Schoen, 2015; Gong and Xue, 2020) to promote the generation of high PI.

(2) A2: PI*AM*SE*IM

As long as the IM of the organization is perfect, the internal driving forces of PI, AM, and SE can be stimulated to produce a comprehensive effect, which will induce the career growth behavior of GPs. The perfect IM is the external force for career growth. The formulation of regulations can fully mobilize the enthusiasm of GPs mainly from the material (promotion opportunities) and spirit (awarding medals) perspectives to meet their motivational needs and form a strong sense of happiness and belonging to produce a high PI (Wang et al., 2016). The higher the PI is, the stronger the goals, initiative, and persistence to pursue success. Therefore, GPs can respond positively and constantly seek solutions until the goal is achieved when facing difficulties and challenges.

(3) A3: PI*∼AM*∼E*∼IM*∼TMs

Regardless of whether AM is clear, SE is strong, and the IM and TM are perfect, as long as GPs have a high PI, which will lead to career growth behavior. PI is the individual recognition of the nature and content of one’s occupation, and it is the individual basis for working hard and achieving organizational goals. With the intensification of medical system reform in China, GPs shoulder the responsibility of building a comprehensive and responsible health medical management mode of the “integration of first diagnosis and referral.” PI is the internal driving force for individuals to realize their life value. The higher the PI is, the stronger the desire to achieve the established goals. It is possible to improve GPs’ professional ability in various ways, accelerating their career growth.

By comparing the coverage indexes of the three configurations, it can be seen that A2 has the highest case coverage, which can explain 80% of the result variable. Therefore, it is more likely to produce high career growth of GPs, indicating most GPs obtain high career growth *via* the pathway. This fully shows that high PI, AM, and SE are the internal driving forces for GPs to achieve high career growth. These positive psychological factors serve as a solid bulwark against real problems such as low wages and distrust among residents. Meanwhile, the high IM can fully mobilize the enthusiasm of GPs. By formulating contract service policies, the government increases the income level of GPs. In addition, to broaden their career development pathways, specific posts are set up to encourage GPs to work at the grass-roots level so that their professional and managerial abilities can be fully trained. Therefore, according to the interaction of internal and external factors, it is easier to stimulate the high career growth of GPs.

Through a comprehensive analysis of the three configurations, PI as a core condition can directly affect the generation of high career growth of GPs in the A3 pathway. Due to the low skill level of GPs, they have not been trusted by residents. Low social status recognition makes them doubt their value and leads to job burnout. In recent years, China has gradually affirmed the importance of GPs, believing that they are “residents’ health gatekeepers,” and has begun to cultivate a large number of qualified GP’s and establish a perfect team of GPs, to establish the social status of GPs and increase their PI. Meanwhile, it is found that there is a substitution effect of PI, AM, and SE in A1 and A2 pathways. When high IM existence or high IM and high TM simultaneous absence, it will produce high career growth. As a positive psychological factor, high PI, high SE, and high AM in pathways A1 and A2 are the internal driving forces affecting behaviors, which can effectively mobilize the subjective initiative and thus stimulate high career growth of GPs. China improves the skills of GPs to obtain high AM and SE. At the same time, it can improve the salary level to obtain high PI. Thus, the comprehensive effect of the three factors on high career growth is increased.

### Not-High Career Growth Configurations of General Practitioners

There are two configurations, including NA1 and NA2, that can produce not-high career growth. Firstly, NA1 shows that whether PI exists or not, as long as lacking AM, SE, IM, and TM, GPs will not produce a high level of career growth. Secondly, NA2 shows that as long as lacking TM, even if there is a high PI, AM, and SE, GPs will not produce a high level of career growth. Lastly, through comprehensive analysis, it can be seen that NA1 and NA2 all show the same characteristics; that is, GPs’ career growth is inseparable from the organization to develop a sound TM. As long as lacking TM, it will lead to not-high career growth. In recent years, China has successfully implemented the TM for GPs in Jiangsu and Guizhou Province. According to different GPs, various training strategies were formulated, such as continuing education and job transfer training, which can effectively improve their professional abilities to avoid not-high career growth of GPs.

## Conclusion

In our study, 407 GPs from different regions in China were selected as samples. From the perspectives of psychology and organization, we constructed five antecedent conditions and used configurational theorizing and QCA to explore these multiple drivers of high career growth. The conclusions are as follows. The study found that there are three pathways of high career growth. The three interactive internal driving forces promote the development of high career growth, forming the pathway A1. The IM, as an external force, acts on the internal driving force to stimulate the comprehensive effect of the three elements and can promote its adjustment and improvement at the same time. The linkage matching of internal and external factors to jointly promote GPs’ high career growth, forming the pathway A2. The development of career growth is based on the understanding and recognition of the occupation, which directly affects the degree of goal setting and effort, forming the pathway A3. The A2 path explains 80% of the resulting variable. Therefore, it is the optimal path to produce high career growth for GPs. The pathways show that PI has a direct impact on high career growth in A3. Pathway A1 shows the comprehensive effect of three internal driving forces (PI, AM, and SE) to stimulate high career growth at the psychological level. Pathway A2 is formed by the interaction of psychological factors and organizational factors.

### Theoretical Contribution

This study selected five key antecedent conditions at the psychological and organizational levels to investigate the driving mechanism of career growth for GPs. The previous studies were limited to the psychological or organizational level, but the internal mechanism of the synergistic effect from the two levels on career growth was unclear. Therefore, this study enriched psychological level findings by examining the organizational level and deeply analyzing the collaborative mechanism. This study not only found three pathways that affect career growth but also found a more efficient pathway. Simultaneously, it is emphasized that the career growth of GPs is not determined by a single factor but depends on the different antecedents of psychological and organizational factors. When explaining the inconsistency of SE on career growth conclusions, we may also consider the matching situation of other factors at the psychological level and organizational level or other factors, which is of great significance to improve the situation of inconsistent career growth research results due to the neglect of multiple factors in previous studies.

In terms of research method, previous regression analysis methods emphasized the net effect of a single factor on career growth but ignored the influence of interactions between and among different antecedent conditions. In this study, we used the QCA method to construct the configuration that influences career growth by integrating antecedent conditions, identifying the pathways and mechanisms of career growth through the influence of multiple concurrent factors, exploring the relationship among pathways, and analyzing mutual substitutions for different conditions.

In terms of methodology, we have reversed the previous situation which paying attention to the study of symmetry and ignoring the study of asymmetry. In our study, the asymmetric set relationship is used to replace the correlation relationship, and the causal complexity affecting the career growth of GPs is explained from the perspective of set theory.

### Managerial Implications

To strengthen the relationship among the internal driving forces of career growth, it is necessary to improve the PI, AM, and SE of GPs. The stimulation of high AM and SE comes from the devotion and recognition of their occupation, but lower social recognition leads to lower PI. Therefore, it is significant to establish GPs’ social image and improve their salary to stimulate high PI to form high AM and SE and achieve the best comprehensive effect.

It is very important to improve the IM and TM. By adjusting the structure and content from two aspects of material and spiritual incentives, the IM can consistently satisfy the conditions that can fully mobilize the enthusiasm of GPs, such as improving the performance evaluation system, increasing learning opportunities, and awarding honorary titles. The optimization of the TM should involve constant updates to content, such as paying attention to the fairness of assessment and the rationality of training design. The IM and TM are the effective external conditions that ensure the fundamental development of GPs’ career growth (Harris et al., 2020).

Promoting the interaction between external forces and internal driving forces is the decisive factor in the career growth of GPs. The optimization of external forces is the premise that activates the internal driving forces to achieve comprehensive effects. The psychological factors can constantly be enhanced after the internal driving force is influenced by the perfect external forces. In addition, high internal driving forces interact with each other, which can form a chain of mutual influence to stimulate the perfection and optimization of external forces. Therefore, we explain the causal complexity affecting the career growth of GPs from the perspective of set theory, and use configuration thinking to construct the combination of antecedents and conditions. According to this theoretical basis, the external force and internal driving force exert a reciprocal and circular influence to ensure the sustainable and orderly development of the career growth of GPs, so as to implement the policy of “serious diseases into the hospital and minor diseases into the community,” so that medical and health resources can better serve people’s health.

### Limitations and Future Research

This research with the five antecedent conditions for the career growth of GPs still has the following deficiencies. (1) Because the questionnaire respondents were from many different regions, the proportions of questionnaires returned from different regions were uneven. The research results may reflect problems in areas where the proportion of respondents was relatively small. (2) This study focused on the influence of psychological and organizational factors on the career growth of GPs. In the future, we will further study the impact of other factors at the psychological and organizational levels or the societal level on GPs’ career growth.

## Data Availability Statement

The original contributions presented in the study are included in the article/Supplementary Material, further inquiries can be directed to the corresponding authors.

## Ethics Statement

The studies involving human participants were reviewed and approved by the Ethics Committee of Jiangsu University, China. The patients/participants provided their written informed consent to participate in this study.

## Author Contributions

ZM contributed to the conception and research plan of the study. BG was responsible for data collection and analysis and manuscript writing. ML conducted research methodology guidance. ZL helped in writing the manuscript and editing. LY helped collect data. TL helped adjust the structure of the article and put forward guidance suggestions. All authors listed have made a substantial, direct, and intellectual contribution to the work, and approved it for publication.

## Conflict of Interest

The authors declare that the research was conducted in the absence of any commercial or financial relationships that could be construed as a potential conflict of interest.

## Publisher’s Note

All claims expressed in this article are solely those of the authors and do not necessarily represent those of their affiliated organizations, or those of the publisher, the editors and the reviewers. Any product that may be evaluated in this article, or claim that may be made by its manufacturer, is not guaranteed or endorsed by the publisher.
