# Supplementary material for: Influential Pathways of Employees’ Career Growth: Linkage of Psychological and Organizational Factors Based on Qualitative Comparative Analysis
Source: Front Psychol. 2022 Jan 4;12:796454. doi: 10.3389/fpsyg.2021.796454 (PMC8763967; doi:10.3389/fpsyg.2021.796454)
Supplement: Supplementary file 1 [file Table_1.DOCX]

**Table S1** Truth table

| Professional identity  （PI） | Achievement motivation  (AM) | Self-  efficacy  (SE) | Incentive mechanism  (IM) | Training mechanism  (TM) | Number | Career growth | Raw consistency | PRI |
| --- | --- | --- | --- | --- | --- | --- | --- | --- |
| 1 | 1 | 1 | 1 | 1 | 138 | 1 | 0.871 | 0.767 |
| 1 | 1 | 1 | 1 | 0 | 4 | 1 | 0.884 | 0.583 |
| 1 | 1 | 1 | 0 | 0 | 2 | 1 | 0.882 | 0.521 |
| 1 | 0 | 0 | 0 | 0 | 4 | 1 | 0.854 | 0.437 |
| 0 | 0 | 0 | 0 | 0 | 145 | 0 | 0.507 | 0.126 |
